# Accidental High-dose Intrathecal Treatment: Late Results of a Patient

**DOI:** 10.4274/tjh.galenos.2019.2019.0283

**Published:** 2020-02-20

**Authors:** Tiraje Celkan, Evrim Çifçi Sunamak

**Affiliations:** 1 İstanbul University-Cerrahpaşa Cerrahpaşa Faculty of Medicine, Department of Pediatric Hematology Oncology, İstanbul, Turkey; 2 Dr. Lütfi Kırdar Kartal Training and Research Hospital, Child Health and Diseases, İstanbul, Turkey

**Keywords:** Overdose, Intrathecal methotrexate, Folinic acid

## To the Editor,

The increase in reporting accidental and overdose use of childhood treatments, starting from 2014, is promising. Understanding the dynamics of overdose may help in the development of more effective prevention and control strategies [1]. Herein, we want to share one of our patients with an overdose of intrathecal treatment.

The survival rates of patients with leukemia and lymphoma have improved after prophylactic intrathecal methotrexate treatments. There have also been some case reports about the mortality or morbidity of intrathecal methotrexate or folinic acid [2,3,4,5,6,7,8,9]. We experienced a nursing error in which 35 mg of intrathecal methotrexate was administered instead of the planned 12 mg to a 3-year-old boy with T-cell non-Hodgkin lymphoma, stage IV. The patient was receiving the first day of the regimen of the second cytarabine block of protocol I, phase 2, BFM (Berlin-Frankfurt-Münster). The error was recognized after about 60 minutes. We performed neither CSF drainage nor exchange as Kazancı et al. [9] had done for their two patients. We thought of intrathecal folinic acid administration, but there were no data about its intrathecal use. Carboxypeptidase G2 was not available in our country. We administered 9 mg of folinic acid (15 mg/m^2^) intravenously, followed by 100 mg of folinic acid (150 mg/m^2^) infused in 6 hours. The patient was monitored for toxic signs and symptoms. He developed no clinical signs. Cranial computed tomography (CT) performed 2 days after the incident revealed no morphological changes. He was followed after this accident for 15 years. Although his neurological development was normal and his most recent cranial CT and electroencephalography results revealed no sequelae, he has had two unsuccessful suicide attempts.

If a readily available source of information regarding the action needed to be taken after such an incident had been available, we could have approached the child more comfortably and confidently, and our colleagues who used intrathecal folinic acid would not have done so [4]. As methotrexate doses of up to 500 mg have not been associated with untoward events, deciding when to hold an intervention is important.
